# Synthesis and structure of 4-bromo-2-chloro­phenyl 4′-meth­oxy-[1,1′-biphen­yl]-4-carboxyl­ate featuring short halogen⋯oxygen contacts

**DOI:** 10.1107/S2056989025002658

**Published:** 2025-04-04

**Authors:** B. S. Palakshamurthy, H. Anil Kumar, H. C. Devarajegowda, H.T. Srinivasa, M. Harish Kumar

**Affiliations:** a Department of PG Studies and Research in Physics Albert Einstein Block UCS Tumkur University, Tumkur Karnataka-572103 India; b Department of Physics, Government First Grade College, Chikkballapur, Karnataka-562101, India; c Department of Physics Yuvaraja's College University of Mysore,Mysore- 570005 Karnataka India; d Raman Research Institute, C V Raman Avenue Sadashivanagar Bangalore-560080 Karnataka India; University of Aberdeen, United Kingdom

**Keywords:** crystal structure, biphen­yl, Hirshfeld surface

## Abstract

In the title compound, C_20_H_14_BrClO_3_, the dihedral angles between the aromatic ring of the 4-bromo-2-chloro­phenyl and the immediate neighbour and second aromatic ring of the biphenyl moiety are 80.59 (2) and 75.42 (2)°, respectively. The dihedral angle between the rings within the biphenyl moiety is 24.57 (4)°. The torsion angle associated with the ester group linking the biphenyl ring and the 4-bromo-2-chloro­phenyl group is −166.6 (2)°. The extended structure features short halogen⋯oxygen contacts [Cl⋯O = 2.991 (3), Br⋯O = 3.139 (2) Å], forming mol­ecular sheets lying parallel to (101). The Hirshfeld surface analysis reveals that the major contributions to the inter­molecular inter­actions are from C⋯H/H⋯C (32.2%), H⋯H/H⋯H (26.3%), Br⋯H/H⋯Br (10.7%), O⋯H/H⋯O (10.4%) and Cl⋯H/H⋯Cl (7.5%) contacts.

## Abstract 2

The extended structure features short halogen⋯oxygen contacts [Cl⋯O = 2.991 (3), Br⋯O = 3.139 (2) Å], forming mol­ecular sheets lying parallel to (101).

## 1. Chemical context

Biphenyl derivates exhibit medicinal properties such as anti­hypertensive (Sharma *et al.*, 2010 ▸), anti-diabetic (Sachan *et al.*, 2009 ▸), anti-bacterial (Trivedi *et al.*, 2009 ▸), anti­fungal (Zhao *et al.*, 2017 ▸) and anti­cancer (Mukherjee *et al.*, 2016 ▸) effects. The drug obtained from the piperidine derivative of biphenyl-4-carboxyl­ate selectively kills the bacterial persisters that are resistant to anti­biotic treatments (Kim *et al.*, 2011 ▸). Some biphenyl-carb­oxy­lic acid derivatives act as anti­resorptive drugs (Van’t Hof *et al.*, 2004 ▸; Idris *et al.*, 2009 ▸) by stopping or slowing down bone loss in osteoporosis. It is found that biphenyl compounds substituted with a heterocyclic ring can act as anti-tyrosinase agents (Kwong *et al.*, 2017 ▸) that reduce the activity of tyrosinase enzyme. Biphenyl-4-carb­oxy­lic acid derivatives inhibit tubulin polymerization to act as anti­cancer agents (Mahale *et al.*, 2014 ▸). 4-Bromo-2-chloro­phenyl-based compounds exhibit significant *in vitro* inhibitory effects on plasmodium falciparum against malaria parasites (Vallone *et al.*, 2018 ▸, Kos *et al.*, 2022 ▸). The presence of a halogen atom in the phenyl moiety of 4-bromo-2-chloro­phenyl derivatives is found to induce anti­microbial properties in the compounds (Radwan *et al.*, 2014 ▸).
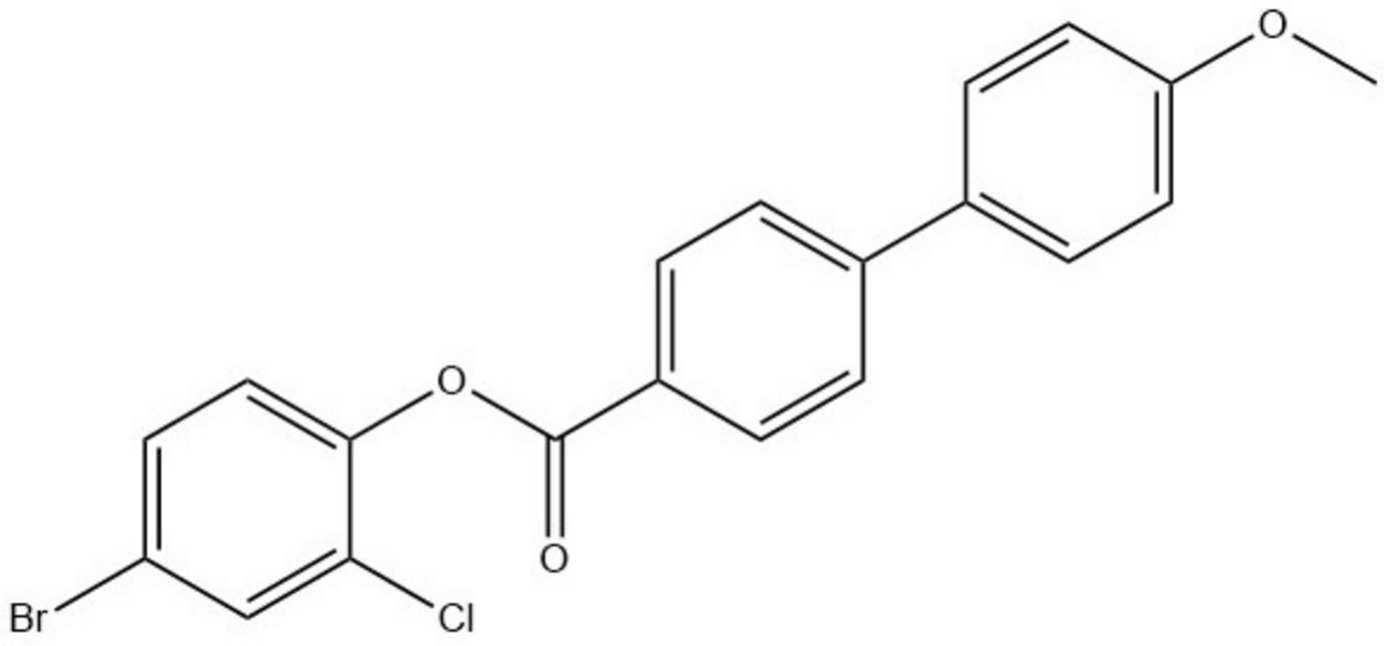


As part of our studies in this area, we now present the synthesis and crystal structure of the title compound, C_20_H_14_BrClO_3_ (**I**).

## 2. Structural commentary

**Figure 1:**
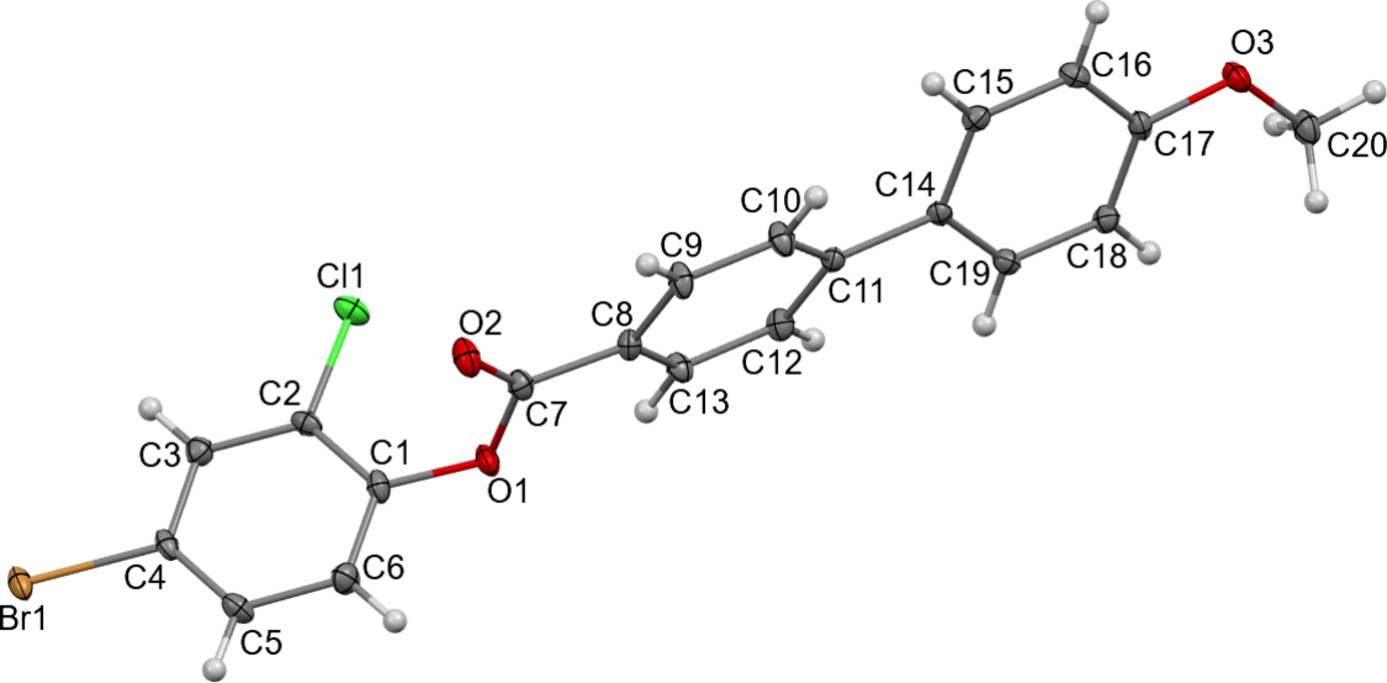
The mol­ecular structure of (**I**) showing displacement ellipsoids drawn at the 50% probability level.

The mol­ecular structure of (**I**) is shown in Fig. 1 ▸. The dihedral angle between the aromatic ring (C1–C6) of the 4-bromo-2-chloro­phenyl group and the C8–C13 and C14–C19 rings of the meth­oxy-biphenyl-carboxyl­ate moiety are 80.59 (2) and 75.42 (2)°, respectively. The dihedral angle between the aromatic rings in the biphenyl moiety (C8–C13 and C14–C19) is 24.57 (4)°. The torsion angle in the ester group (C1—O1—C7—C8) linking the 4-bromo-2-chloro­phenyl group with the biphenyl moiety is −166.6 (2)°.

## 3. Supra­molecular features

**Figure 2:**
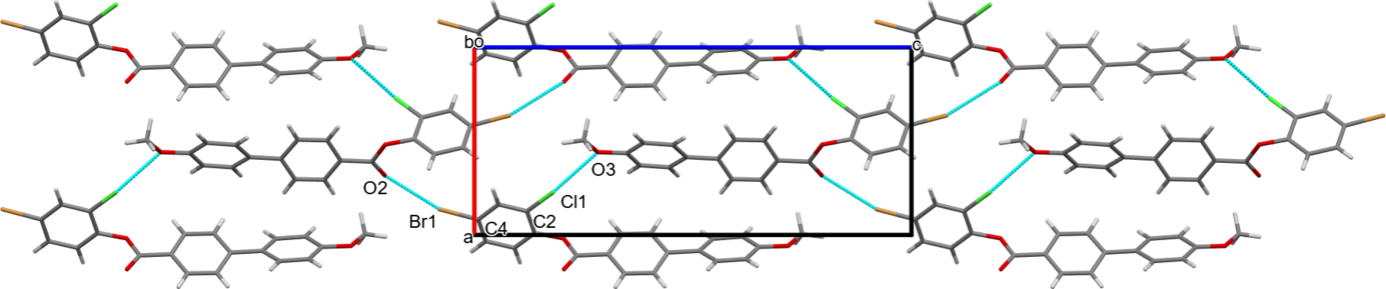
The packing of (**I**) with dashed lines indicating Cl⋯O and Br⋯O contacts.

The crystal packing features short C2—Cl1⋯O3 and C4—Br1⋯O2 inter­actions with a Cl1⋯O3 distance of 2.991 (3) Å and a Br1⋯O2 distance of 3.139 (2) Å, forming mol­ecular sheets propagating in the (101) plane, as shown in Fig. 2 ▸. The van der Waals separations of Cl and O atoms and Br and O atoms are 3.27 and 3.37 Å, respectively. Two weak C—H⋯π inter­actions also occur (Table 1 ▸).

## 4. Hirshfeld surface analysis

**Figure 3:**
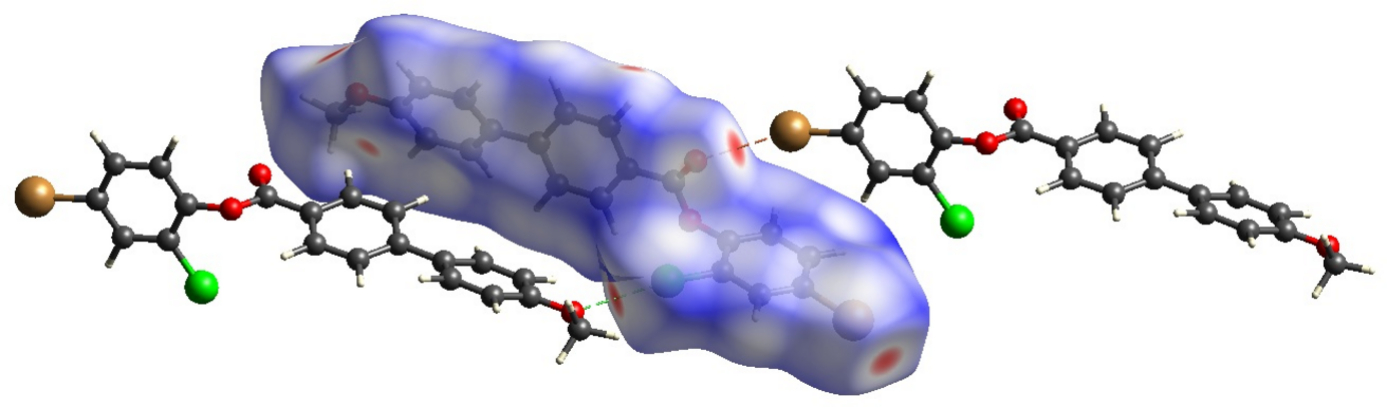
The Hirshfeld surface of (**I**) mapped over *d_norm_* with red spots corresponding to Cl⋯O and Br⋯O short contacts.

**Figure 4:**
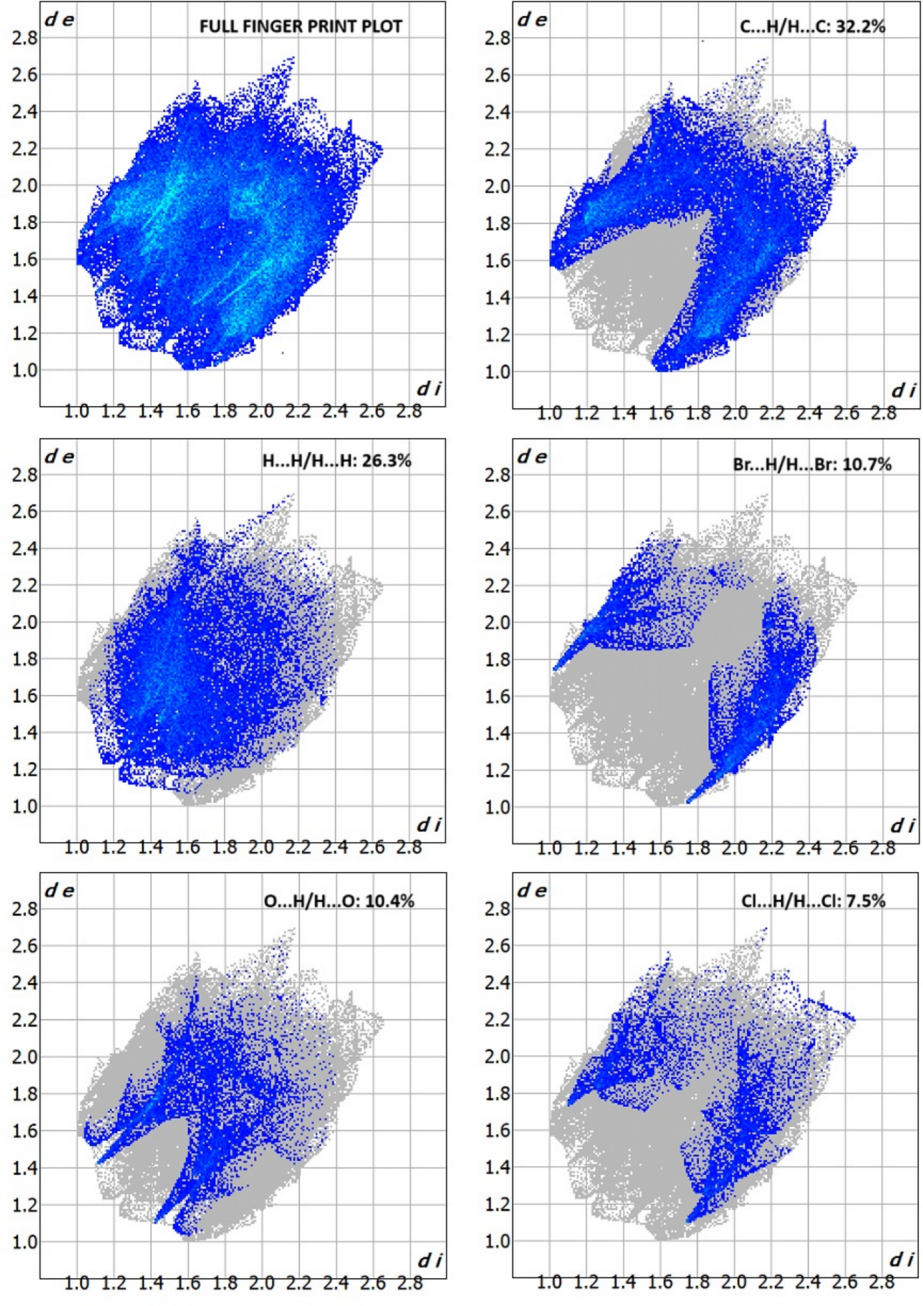
The two-dimensional fingerprint plots of the major contributors to inter­molecular inter­actions in (**I**).

**Figure 5:**
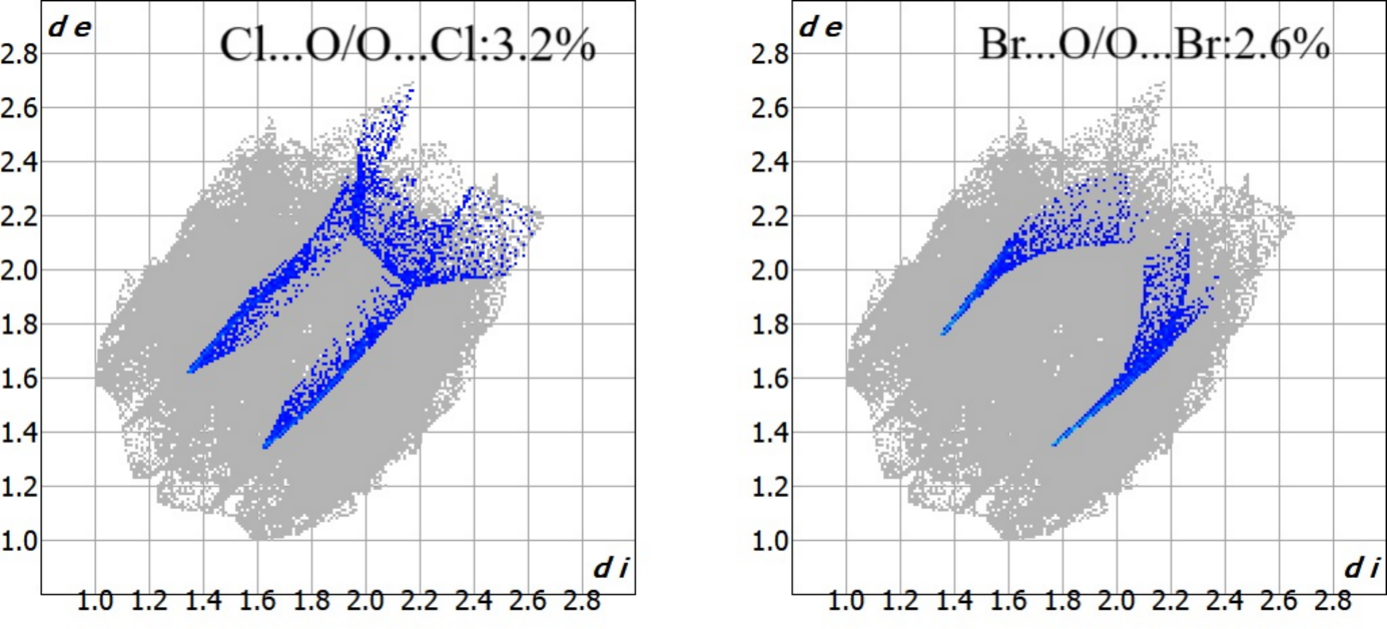
Fingerprints plots for the Cl⋯O/O⋯Cl and Br⋯O/O⋯Br contacts in (**I**).

A Hirshfeld surface analysis for (**I**) was performed to qu­antify and visualize the inter­molecular inter­action present in the mol­ecules using *Crystal-Explorer17* (Turner *et al.*, 2017 ▸). The Hirshfeld surface (Spackman & Jayatilaka, 2009 ▸) mapped over normalised contact distance *d*_norm_ (Fig. 3 ▸) shows the presence of red spots on the iso-surface that correspond to the existence of the short halogen⋯oxygen type inter­actions noted above. The two-dimensional fingerprint plots (McKinnon *et al.*, 2007 ▸) are shown in Fig. 4 ▸. The major contributions for the inter­molecular inter­actions are from C⋯H/H⋯C (32.2%), H⋯H (26.3%), Br⋯H/H⋯Br (10.7%), O⋯H/H⋯O (10.4%) and Cl⋯H/H⋯Cl (7.5%). The sharp spikes in the fingerprint plots for Cl⋯O and Br⋯O contacts (Fig. 5 ▸) confirm the existence of the directional halogen⋯oxygen inter­actions.

## 5. Database survey

A search of the Cambridge Structural Database (CSD version 2.0.4, December 2019; Groom *et al.*, 2016 ▸) for mol­ecules containing a [1,1′-biphen­yl]-4-carboxyl­ate fragment resulted in more than thirty matches, but six compounds were identified with a substitution at the oxygen atom of the ester group similar to the title compound. In five of the compounds, namely CSD refcodes PUGZUP (Chen *et al.*, 2020 ▸), ESEMAT (Wang *et al.*, 2021 ▸), FIRYIR(Royal *et al.*, 2019 ▸), JOCVAB (Chen *et al.*, 2019 ▸) and JOCVEF (Chen *et al.*, 2019 ▸), the dihedral angles between the aromatic rings of the biphenyl carb­oxy­lic acid range between 29.42 (2) and 38.39 (3)° whereas in NEKPAK (Wang *et al.*, 2017 ▸), the dihedral angle is 12.42 (2)°. The conformations of the ester groups (C—O—C—C), which link the biphenyl ring and the functional group in the above compounds and (**I**), are all *anti*.

## 6. Synthesis and crystallization

A mixture of 4-bromo-2-chloro­phenol (0.208 g, 1.00 mmol) and 4′-meth­oxy-[1,1′-biphen­yl]-4-carb­oxy­lic acid (0.228 g, 1.00 mmol) was suspended in anhydrous chloro­form (10 ml). To this were added *N*,*N*-di­cyclo­hexyl­carbodi­imide (0.206 g, 1.00 mmol) and 4-*N*,*N*-di­methyl­amino pyridine (5 mg) and the mixture was stirred overnight at room temperature. The *N*,*N*-di­cyclo­hexyl urea formed was filtered off and the filtrate diluted with chloro­form (25 ml). This solution was washed successively with 5% aqueous acetic acid solution (2 × 25 ml) and water (2 × 25 ml) and dried over sodium sulfate. The residue obtained on removal of the solvent was chromatographed on silica gel using chloro­form as the eluent. Removal of solvent from the eluate afforded a white material, which was recrystallized from the mixed solvents of chloro­form and petroleum ether to yield colourless prisms of (**I**). Yield 78%. Elemental analysis calculated: C, 57.51; H, 3.38; Br, 19.13; Cl, 8.49; O, 11.49% found is C, 57.56; H, 3.39; Br, 19.15; Cl, 8.53%, m.p. 338–340 K.

## 7. Refinement

Crystal data, data collection and structure refinement details are summarized in Table 2 ▸. All H atoms were positioned with idealized geometry and refined using a riding model with C—H = 0.93–0.96 Å and *U*_iso_(H) = 1.2*U*_eq_(C) or 1.5*U*_eq_(methyl C).

## Supplementary Material

Crystal structure: contains datablock(s) I. DOI: 10.1107/S2056989025002658/hb8126sup1.cif

Structure factors: contains datablock(s) I. DOI: 10.1107/S2056989025002658/hb8126Isup2.hkl

Supporting information file. DOI: 10.1107/S2056989025002658/hb8126Isup3.cml

CCDC reference: 2433444

Additional supporting information:  crystallographic information; 3D view; checkCIF report

## Figures and Tables

**Table 1:** Hydrogen-bond geometry (Å, °) *Cg*3 is the centroid of the C14–C19 ring.

*D*—H⋯*A*	*D*—H	H⋯*A*	*D*⋯*A*	*D*—H⋯*A*
C6—H6⋯*Cg*3^i^	0.93	2.65	3.459 (3)	147
C10—H10⋯*Cg*3^ii^	0.93	2.85	3.577 (3)	136

Symmetry codes: (i) 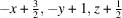; (ii) 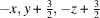.

**Table 2:** Experimental details

Crystal data	
Chemical formula	C_20_H_14_BrClO_3_
*M*_r_	417.67
Crystal system, space group	Orthorhombic, *P*2_1_2_1_2_1_
Temperature (K)	296
*a*, *b*, *c* (Å)	8.8347 (4), 9.4124 (5), 20.5526 (11)
*V* (Å^3^)	1709.07 (15)
*Z*	4
Radiation type	Mo *K*α
μ (mm^−1^)	2.58
Crystal size (mm)	0.40 × 0.35 × 0.29

Data collection	
Diffractometer	Bruker SMART APEXII CCD
Absorption correction	Multi-scan (*SADABS*; Krause *et al.*, 2015 ▸)
*T*_min_, *T*_max_	0.371, 0.475
No. of measured, independent and observed [*I* > 2σ(*I*)] reflections	28805, 4264, 4012
*R*_int_	0.053
(sin θ/λ)_max_ (Å^−1^)	0.668

Refinement	
*R*[*F*^2^ > 2σ(*F*^2^)], *wR*(*F*^2^), *S*	0.023, 0.054, 1.04
No. of reflections	4264
No. of parameters	228
H-atom treatment	H-atom parameters constrained
Δρ_max_, Δρ_min_ (e Å^−3^)	0.27, −0.23
Absolute structure	Flack parameter
Absolute structure parameter	0.011 (8)

Computer programs: *APEX2* and *SAINT* (Bruker, 2017 ▸), *SHELXL2018/3* and *SHELXL2019/2* (Sheldrick, 2015*b* ▸), *SHELXT2018/2* (Sheldrick, 2015*a* ▸) and *Mercury* (Macrae *et al.*, 2020 ▸).

## supplementary crystallographic information

### 4-Bromo-2-chlorophenyl 4'-methoxy-[1,1'-biphenyl]-4-carboxylate Crystal data

**Table:**

C_20_H_14_BrClO_3_	prism
*M**_r_* = 417.67	*D*_x_ = 1.623 Mg m^−^^3^
Orthorhombic, *P*2_1_2_1_2_1_	Mo *K*α radiation, λ = 0.71073 Å
*a* = 8.8347 (4) Å	Cell parameters from 4264 reflections
*b* = 9.4124 (5) Å	θ = 2.5–28.5°
*c* = 20.5526 (11) Å	µ = 2.58 mm^−^^1^
*V* = 1709.07 (15) Å^3^	*T* = 296 K
*Z* = 4	Prism, colourless
*F*(000) = 840	0.40 × 0.35 × 0.29 mm

### 4-Bromo-2-chlorophenyl 4'-methoxy-[1,1'-biphenyl]-4-carboxylate Data collection

**Table:**

Bruker SMART APEXII CCD diffractometer	4264 independent reflections
Radiation source: fine-focus sealed tube	4012 reflections with *I* > 2σ(*I*)
Graphite monochromator	*R*_int_ = 0.053
Detector resolution: 1.02 pixels mm^-1^	θ_max_ = 28.4°, θ_min_ = 2.0°
ω scans	*h* = −11→11
Absorption correction: multi-scan (SADABS; Krause *et al.*, 2015)	*k* = −12→11
*T*_min_ = 0.371, *T*_max_ = 0.475	*l* = −27→27
28805 measured reflections	

### 4-Bromo-2-chlorophenyl 4'-methoxy-[1,1'-biphenyl]-4-carboxylate Refinement

**Table:**

Refinement on *F*^2^	Secondary atom site location: difference Fourier map
Least-squares matrix: full	Hydrogen site location: inferred from neighbouring sites
*R*[*F*^2^ > 2σ(*F*^2^)] = 0.023	H-atom parameters constrained
*wR*(*F*^2^) = 0.054	*w* = 1/[σ^2^(*F*_o_^2^) + (0.0209*P*)^2^ + 0.2511*P*] where *P* = (*F*_o_^2^ + 2*F*_c_^2^)/3
*S* = 1.04	(Δ/σ)_max_ = 0.005
4264 reflections	Δρ_max_ = 0.27 e Å^−^^3^
228 parameters	Δρ_min_ = −0.23 e Å^−^^3^
0 restraints	Absolute structure: Flack parameter
0 constraints	Absolute structure parameter: 0.011 (8)
Primary atom site location: dual	

### 4-Bromo-2-chlorophenyl 4'-methoxy-[1,1'-biphenyl]-4-carboxylate Special details

**Table:**

Geometry. All esds (except the esd in the dihedral angle between two l.s. planes) are estimated using the full covariance matrix. The cell esds are taken into account individually in the estimation of esds in distances, angles and torsion angles; correlations between esds in cell parameters are only used when they are defined by crystal symmetry. An approximate (isotropic) treatment of cell esds is used for estimating esds involving l.s. planes.
Refinement. Refined as a 2-component inversion twin.

### 4-Bromo-2-chlorophenyl 4'-methoxy-[1,1'-biphenyl]-4-carboxylate Fractional atomic coordinates and isotropic or equivalent isotropic displacement parameters (Å^2^)

**Table:**

	*x*	*y*	*z*	*U*_iso_*/*U*_eq_	
Br1	0.36294 (3)	0.65415 (3)	1.07977 (2)	0.02179 (8)	
Cl1	0.27206 (8)	0.77586 (8)	0.82023 (3)	0.02310 (15)	
O1	0.5178 (2)	0.5732 (2)	0.79614 (9)	0.0205 (4)	
O2	0.6829 (2)	0.7562 (2)	0.79511 (9)	0.0237 (4)	
O3	0.5585 (2)	0.6213 (2)	0.28240 (9)	0.0200 (4)	
C1	0.4892 (3)	0.5971 (3)	0.86220 (13)	0.0170 (5)	
C2	0.3733 (3)	0.6880 (3)	0.87978 (12)	0.0162 (5)	
C3	0.3367 (3)	0.7066 (3)	0.94475 (13)	0.0179 (5)	
H3	0.260073	0.768730	0.957100	0.021*	
C4	0.4174 (3)	0.6301 (3)	0.99101 (12)	0.0163 (5)	
C5	0.5330 (3)	0.5395 (3)	0.97413 (13)	0.0192 (6)	
H5	0.586627	0.490548	1.005954	0.023*	
C6	0.5685 (3)	0.5224 (3)	0.90840 (13)	0.0200 (6)	
H6	0.645518	0.460749	0.895960	0.024*	
C7	0.6090 (3)	0.6712 (3)	0.76569 (12)	0.0173 (5)	
C8	0.6024 (3)	0.6556 (3)	0.69402 (12)	0.0162 (5)	
C9	0.7052 (3)	0.7323 (3)	0.65671 (13)	0.0193 (5)	
H9	0.776532	0.789750	0.677077	0.023*	
C10	0.7022 (3)	0.7237 (3)	0.58936 (13)	0.0199 (5)	
H10	0.772926	0.773917	0.565012	0.024*	
C11	0.5936 (3)	0.6401 (3)	0.55758 (12)	0.0137 (5)	
C12	0.4918 (3)	0.5638 (3)	0.59588 (12)	0.0181 (6)	
H12	0.419557	0.507014	0.575668	0.022*	
C13	0.4953 (3)	0.5702 (3)	0.66334 (13)	0.0171 (5)	
H13	0.426611	0.517802	0.687867	0.021*	
C14	0.5846 (3)	0.6327 (3)	0.48544 (12)	0.0137 (5)	
C15	0.6389 (3)	0.7435 (3)	0.44582 (12)	0.0163 (5)	
H15	0.682792	0.822772	0.465137	0.020*	
C16	0.6283 (3)	0.7369 (3)	0.37886 (12)	0.0172 (5)	
H16	0.664628	0.811468	0.353630	0.021*	
C17	0.5634 (3)	0.6189 (3)	0.34898 (12)	0.0160 (5)	
C18	0.5083 (3)	0.5085 (3)	0.38685 (12)	0.0163 (5)	
H18	0.464090	0.429602	0.367308	0.020*	
C19	0.5197 (3)	0.5164 (3)	0.45424 (13)	0.0158 (5)	
H19	0.482771	0.441679	0.479239	0.019*	
C20	0.4945 (4)	0.5005 (3)	0.25032 (13)	0.0238 (6)	
H20A	0.509264	0.509222	0.204215	0.036*	
H20B	0.388077	0.495699	0.259587	0.036*	
H20C	0.543054	0.415659	0.265624	0.036*	

### 4-Bromo-2-chlorophenyl 4'-methoxy-[1,1'-biphenyl]-4-carboxylate Atomic displacement parameters (Å^2^)

**Table:**

	*U*^11^	*U*^22^	*U*^33^	*U*^12^	*U*^13^	*U*^23^
Br1	0.03127 (14)	0.02241 (13)	0.01170 (11)	−0.00941 (12)	0.00389 (11)	−0.00150 (11)
Cl1	0.0232 (3)	0.0267 (3)	0.0194 (3)	0.0006 (3)	−0.0048 (3)	0.0066 (3)
O1	0.0279 (10)	0.0228 (10)	0.0107 (9)	−0.0050 (8)	0.0038 (8)	0.0002 (8)
O2	0.0236 (10)	0.0321 (11)	0.0153 (9)	−0.0082 (9)	0.0012 (8)	−0.0034 (8)
O3	0.0248 (10)	0.0231 (11)	0.0120 (9)	−0.0039 (8)	−0.0019 (8)	0.0016 (7)
C1	0.0160 (12)	0.0222 (13)	0.0128 (12)	−0.0036 (11)	0.0030 (10)	−0.0014 (10)
C2	0.0160 (11)	0.0177 (13)	0.0147 (11)	−0.0027 (10)	−0.0046 (10)	0.0035 (9)
C3	0.0180 (13)	0.0174 (12)	0.0182 (12)	−0.0007 (10)	0.0023 (10)	−0.0003 (10)
C4	0.0190 (12)	0.0196 (14)	0.0104 (11)	−0.0076 (11)	0.0023 (9)	0.0008 (10)
C5	0.0181 (13)	0.0243 (14)	0.0152 (13)	−0.0012 (12)	−0.0015 (10)	0.0057 (11)
C6	0.0173 (12)	0.0231 (14)	0.0197 (14)	0.0030 (11)	0.0033 (11)	0.0010 (11)
C7	0.0150 (12)	0.0210 (13)	0.0160 (11)	−0.0002 (11)	0.0021 (10)	0.0002 (10)
C8	0.0161 (12)	0.0191 (12)	0.0134 (11)	0.0003 (11)	0.0015 (9)	−0.0029 (11)
C9	0.0179 (12)	0.0258 (14)	0.0142 (12)	−0.0077 (12)	0.0001 (10)	−0.0041 (11)
C10	0.0192 (12)	0.0250 (13)	0.0157 (14)	−0.0067 (11)	0.0034 (11)	0.0000 (11)
C11	0.0143 (11)	0.0141 (12)	0.0127 (11)	0.0015 (10)	−0.0003 (9)	−0.0010 (10)
C12	0.0163 (12)	0.0204 (13)	0.0177 (13)	−0.0028 (11)	−0.0014 (10)	−0.0020 (10)
C13	0.0163 (12)	0.0193 (13)	0.0158 (13)	−0.0041 (11)	0.0031 (10)	0.0017 (10)
C14	0.0124 (11)	0.0160 (13)	0.0128 (11)	0.0036 (10)	−0.0008 (9)	0.0011 (10)
C15	0.0167 (11)	0.0144 (12)	0.0179 (12)	0.0008 (11)	−0.0014 (11)	−0.0019 (9)
C16	0.0173 (11)	0.0176 (12)	0.0168 (12)	−0.0012 (12)	0.0004 (11)	0.0045 (9)
C17	0.0151 (12)	0.0214 (14)	0.0114 (12)	0.0030 (10)	−0.0002 (10)	−0.0008 (9)
C18	0.0177 (12)	0.0152 (13)	0.0162 (13)	−0.0005 (10)	−0.0037 (10)	−0.0018 (10)
C19	0.0161 (12)	0.0160 (13)	0.0154 (12)	0.0010 (11)	−0.0004 (10)	0.0029 (10)
C20	0.0287 (14)	0.0283 (16)	0.0143 (13)	−0.0021 (13)	−0.0031 (11)	−0.0035 (11)

### 4-Bromo-2-chlorophenyl 4'-methoxy-[1,1'-biphenyl]-4-carboxylate Geometric parameters (Å, º)

**Table:**

Br1—C4	1.900 (2)	C10—C11	1.402 (4)
Cl1—C2	1.727 (3)	C10—H10	0.9300
O1—C7	1.375 (3)	C11—C12	1.395 (4)
O1—C1	1.399 (3)	C11—C14	1.487 (3)
O2—C7	1.196 (3)	C12—C13	1.388 (4)
O3—C17	1.369 (3)	C12—H12	0.9300
O3—C20	1.431 (3)	C13—H13	0.9300
C1—C6	1.374 (4)	C14—C19	1.392 (4)
C1—C2	1.382 (4)	C14—C15	1.408 (3)
C2—C3	1.385 (4)	C15—C16	1.381 (3)
C3—C4	1.389 (4)	C15—H15	0.9300
C3—H3	0.9300	C16—C17	1.392 (4)
C4—C5	1.375 (4)	C16—H16	0.9300
C5—C6	1.396 (4)	C17—C18	1.387 (4)
C5—H5	0.9300	C18—C19	1.391 (3)
C6—H6	0.9300	C18—H18	0.9300
C7—C8	1.482 (3)	C19—H19	0.9300
C8—C9	1.391 (4)	C20—H20A	0.9600
C8—C13	1.393 (4)	C20—H20B	0.9600
C9—C10	1.387 (4)	C20—H20C	0.9600
C9—H9	0.9300		
			
C7—O1—C1	116.1 (2)	C11—C10—H10	119.6
C17—O3—C20	117.4 (2)	C12—C11—C10	117.9 (2)
C6—C1—C2	120.9 (2)	C12—C11—C14	120.3 (2)
C6—C1—O1	119.7 (2)	C10—C11—C14	121.9 (2)
C2—C1—O1	119.1 (2)	C13—C12—C11	121.8 (2)
C6—C1—O1	119.7 (2)	C13—C12—H12	119.1
C2—C1—O1	119.1 (2)	C11—C12—H12	119.1
C1—C2—C3	120.2 (2)	C12—C13—C8	119.5 (2)
C1—C2—Cl1	119.6 (2)	C12—C13—H13	120.3
C3—C2—Cl1	120.1 (2)	C8—C13—H13	120.3
C2—C3—C4	118.3 (2)	C19—C14—C15	117.2 (2)
C2—C3—H3	120.8	C19—C14—C11	121.3 (2)
C4—C3—H3	120.8	C15—C14—C11	121.6 (2)
C5—C4—C3	122.0 (2)	C16—C15—C14	121.3 (2)
C5—C4—Br1	120.3 (2)	C16—C15—H15	119.3
C3—C4—Br1	117.7 (2)	C14—C15—H15	119.3
C4—C5—C6	118.9 (3)	C15—C16—C17	120.2 (2)
C4—C5—H5	120.6	C15—C16—H16	119.9
C6—C5—H5	120.6	C17—C16—H16	119.9
C1—C6—C5	119.7 (3)	O3—C17—C18	124.2 (2)
C1—C6—H6	120.2	O3—C17—C16	116.1 (2)
C5—C6—H6	120.2	C18—C17—C16	119.6 (2)
O2—C7—O1	122.5 (2)	C17—C18—C19	119.6 (2)
O2—C7—O1	122.5 (2)	C17—C18—H18	120.2
O2—C7—C8	126.2 (2)	C19—C18—H18	120.2
O1—C7—C8	111.3 (2)	C18—C19—C14	122.1 (3)
O1—C7—C8	111.3 (2)	C18—C19—H19	119.0
C9—C8—C13	119.6 (2)	C14—C19—H19	119.0
C9—C8—C7	118.1 (2)	O3—C20—H20A	109.5
C13—C8—C7	122.3 (2)	O3—C20—H20B	109.5
C10—C9—C8	120.5 (2)	H20A—C20—H20B	109.5
C10—C9—H9	119.8	O3—C20—H20C	109.5
C8—C9—H9	119.8	H20A—C20—H20C	109.5
C9—C10—C11	120.7 (2)	H20B—C20—H20C	109.5
C9—C10—H10	119.6		
			
O1—O1—C1—C6	0.0 (6)	O2—C7—C8—C13	−169.1 (3)
C7—O1—C1—C6	−100.4 (3)	O1—C7—C8—C13	11.1 (4)
O1—O1—C1—C2	0.0 (6)	O1—C7—C8—C13	11.1 (4)
C7—O1—C1—C2	84.4 (3)	C13—C8—C9—C10	−0.3 (4)
C7—O1—C1—O1	0 (34)	C7—C8—C9—C10	−178.8 (3)
C6—C1—C2—C3	0.9 (4)	C8—C9—C10—C11	1.4 (4)
O1—C1—C2—C3	176.0 (2)	C9—C10—C11—C12	−1.5 (4)
O1—C1—C2—C3	176.0 (2)	C9—C10—C11—C14	178.0 (2)
C6—C1—C2—Cl1	−178.0 (2)	C10—C11—C12—C13	0.6 (4)
O1—C1—C2—Cl1	−2.8 (3)	C14—C11—C12—C13	−178.9 (2)
O1—C1—C2—Cl1	−2.8 (3)	C11—C12—C13—C8	0.4 (4)
C1—C2—C3—C4	−1.1 (4)	C9—C8—C13—C12	−0.6 (4)
Cl1—C2—C3—C4	177.7 (2)	C7—C8—C13—C12	177.8 (2)
C2—C3—C4—C5	1.2 (4)	C12—C11—C14—C19	−24.4 (4)
C2—C3—C4—Br1	−178.8 (2)	C10—C11—C14—C19	156.1 (3)
C3—C4—C5—C6	−1.1 (4)	C12—C11—C14—C15	154.5 (3)
Br1—C4—C5—C6	178.9 (2)	C10—C11—C14—C15	−25.0 (4)
C2—C1—C6—C5	−0.7 (4)	C19—C14—C15—C16	−0.1 (4)
O1—C1—C6—C5	−175.8 (2)	C11—C14—C15—C16	−179.1 (3)
O1—C1—C6—C5	−175.8 (2)	C14—C15—C16—C17	−0.2 (4)
C4—C5—C6—C1	0.8 (4)	C20—O3—C17—C18	−1.1 (4)
O1—O1—C7—O2	0.0 (5)	C20—O3—C17—C16	179.1 (2)
C1—O1—C7—O2	13.6 (4)	C15—C16—C17—O3	−179.7 (3)
C1—O1—C7—O1	0 (67)	C15—C16—C17—C18	0.5 (4)
O1—O1—C7—C8	0.0 (4)	O3—C17—C18—C19	179.7 (3)
C1—O1—C7—C8	−166.6 (2)	C16—C17—C18—C19	−0.5 (4)
O2—C7—C8—C9	9.3 (4)	C17—C18—C19—C14	0.2 (4)
O1—C7—C8—C9	−170.5 (2)	C15—C14—C19—C18	0.1 (4)
O1—C7—C8—C9	−170.5 (2)	C11—C14—C19—C18	179.1 (2)

### 4-Bromo-2-chlorophenyl 4'-methoxy-[1,1'-biphenyl]-4-carboxylate Hydrogen-bond geometry (Å, º)

*Cg*3 is the centroid of the C14–C19 ring.

**Table:**

*D*—H···*A*	*D*—H	H···*A*	*D*···*A*	*D*—H···*A*
C6—H6···*Cg*3^i^	0.93	2.65	3.459 (3)	147
C10—H10···*Cg*3^ii^	0.93	2.85	3.577 (3)	136

Symmetry codes: (i) −*x*+3/2, −*y*+1, *z*+1/2; (ii) −*x*, *y*+3/2, −*z*+3/2.

**Table:**

Parameter	SCXRD	DFT	
Br1—C4	1.900 (2)	1.9148	
Cl1—C2	1.727 (3)	1.7468	
O1—C7	1.375 (3)	1.3831	
O1—C1	1.399 (3)	1.3848	
O2—C7	1.196 (3)	1.2025	
O3—C17	1.369 (3)	1.3619	
C7—O1—C1	116.1 (2)	117.908	
C17—O3—C20	117.4 (2)	118.693	
C6—C1—C2	120.9 (2)	119.670	
C6—C1—O1	119.7 (2)	119.844	
O2—C7—O1	122.5 (2)	122.388	
O2—C7—C8	126.2 (2)	126.007	
C1—O1—C7—C8	-166.6 (2	-179.143	
C7—O1—C1—C6	-100.4 (3)	-88.445	
O1—C1—C2—Cl1	-2.8 (3)	-3.598	
